# Courier delivery of antiretroviral therapy: a cohort study of a South African private‐sector HIV programme

**DOI:** 10.1002/jia2.26360

**Published:** 2024-09-18

**Authors:** Yann Ruffieux, Naomi Folb, Anna Grimsrud, Michael Hislop, Liezl Dunn, Eliane Rohner, Anne Maria Namubiru, Chido Chinogurei, Morna Cornell, Mary‐Ann Davies, Matthias Egger, Gary Maartens, Andreas D. Haas

**Affiliations:** ^1^ Institute of Social and Preventive Medicine University of Bern Bern Switzerland; ^2^ Medscheme Cape Town South Africa; ^3^ International AIDS Society Geneva Switzerland; ^4^ Aid for AIDS Management (Pty) Ltd Cape Town South Africa; ^5^ Centre for Infectious Disease Epidemiology and Research School of Public Health University of Cape Town Cape Town South Africa; ^6^ Division of Public Health Medicine School of Public Health University of Cape Town Cape Town South Africa; ^7^ Population Health Sciences Bristol Medical School University of Bristol Bristol UK; ^8^ Division of Clinical Pharmacology Department of Medicine University of Cape Town Cape Town South Africa

**Keywords:** courier ART, viral suppression, private sector, differentiated care, HIV epidemiology, Africa

## Abstract

**Introduction:**

Courier delivery has become a popular antiretroviral therapy (ART) distribution method in some HIV care settings, yet data on ART courier delivery and how it relates to ART outcomes are scarce. We studied the differences in viral suppression rates between individuals from a South African private sector HIV programme receiving ART by courier delivery and those receiving ART through traditional retail dispensing.

**Methods:**

Individuals aged 15 years or older who were actively enrolled in the Aid for AIDS programme between January 2011 and July 2022 were eligible for the analysis. The outcome of interest was viral suppression defined as a viral load (VL) <400 copies per ml. We calculated adjusted odds ratios (OR) for the association between the ART distribution method and viral suppression, comparing those receiving refills through courier pharmacies versus retail dispensing at the time of the VL testing. We used generalized estimating equations to account for repeated VL testing of the same individual. The models were adjusted for age, sex, calendar year, ART regimen, history of mental illness and medical insurance scheme. We computed adjusted ORs for the calendar periods 2011−2013, 2014−2016, 2017−2019, 2020−2022 and overall.

**Results:**

We extracted 442,619 VL measurements from 68,720 eligible individuals, 39,406 (57.3%) were women. The median number of VL measurements per individual was 6 (IQR 3−10). VL suppression was detected in 398,901 (90.1%) tests, and 185,701 (42.0%) of the tests were taken while the individual was receiving ART by courier delivery. Overall, courier delivery was associated with 5% higher odds of viral suppression than retail dispensing (adjusted OR 1.05, 95% CI 1.02−1.08). The strength and direction of this association varied by calendar period, with an adjusted OR of 1.37 (95% CI 1.27−1.48) in 2011−2013 and 1.02 (95% CI 0.97−1.07) in 2020−2022.

**Conclusions:**

Courier delivery of ART is a viable alternative to retail dispensing in the South African private sector, as it was associated with higher viral suppression until 2016 and similar suppression rates in recent years. Further research is needed to investigate the potential benefits and drawbacks of courier delivery of ART in both private and public healthcare settings.

## 1 INTRODUCTION

Despite significant advances in HIV care, ensuring lifelong retention in care, consistent adherence and viral suppression continue to pose significant challenges, especially for young people with HIV, men, and patients with mental health and substance use problems [1, 2, 3]. Frequent clinic visits for medication refills can be burdensome, especially for those who must take leave from work or travel long distances to reach the clinic [4]. Individuals attending antiretroviral therapy (ART) clinics for refills can contribute to overcrowding of healthcare facilities and longer waiting times, which can impact the quality of care and client satisfaction. These challenges can limit access to medication, resulting in poor adherence or treatment interruptions, and compromising HIV treatment outcomes [5, 6].

Differentiated service delivery models for HIV treatment have been developed to reduce the burden associated with medication refills. These include multi‐month scripting, fast‐track refills, community‐based ART delivery and the use of vending machines to dispense ART medication [7, 8, 9, 10, 11]. Another method that has been implemented in South Africa's public and private healthcare sectors and other low‐ and middle‐income countries is the delivery of ART medication by courier pharmacies [12, 13, 14]. To date, there is limited research focusing on the outcomes of individuals receiving ART through this distribution method. A cohort study of the Aid for AIDS (AfA) programme, a large private‐sector ART programme in South Africa, found that courier delivery of ART was associated with significant improvements in CD4 counts, higher viral suppression rates and lower mortality during the period January 2002−July 2011 [14]. A randomized trial in Tanzania found that community delivery of ART in the public healthcare sector led to non‐inferior virological outcomes compared to standard of care [15]. Retrospective studies from the United States and United Kingdom have shown mixed results with some finding home delivery of ART to be associated with better virological outcomes [16, 17] and others finding no difference in virological outcomes with home delivery versus traditional pharmacy dispensing of medication [18, 19].

In this study, we updated previous analyses [14] to investigate the association between courier pharmacy delivery of ART and viral suppression among people living with HIV enrolled in the AfA programme between 2011 and 2022. We hypothesized that courier pharmacy delivery of ART would be associated with improved viral suppression rates compared to retail ART dispensing.

## 2 METHODS

### 2.1 Setting

The private sector AfA programme provides HIV management to individuals enrolled in various medical insurance schemes in South Africa. Participants are treated by private medical practitioners according to national treatment guidelines [20]. Courier pharmacy was introduced for members of the AfA programme in 2004 as a cost‐containment strategy for funders. This service allows AfA members to have their ART delivered directly to their chosen address, providing an alternative to the traditional method of collecting ART from retail pharmacies. Members of all but one medical scheme can choose to obtain medication from either courier or retail pharmacies at no additional cost to the member. The exception is one of the largest schemes managed by AfA: its members are strongly encouraged to opt for courier delivery with a designated service provider. Choosing retail dispensing or courier delivery with a non‐designated service provider under this scheme incurs a co‐payment, a policy that has resulted in only 2.3% of its members selecting the retail dispensing option.

### 2.2 Study design and participants

We conducted a cohort study covering the period 1st January 2011 to 1st July 2022. We included AfA participants on ART and aged 15 years or older with at least one viral load (VL) measurement during the study period. Members of the insurance scheme that incentivized courier pharmacy use by introducing a co‐payment for those opting for retail dispensing were excluded from the analysis. This exclusion was due to the impact of the co‐payment on members’ choice, which compromises the comparability of the exposure groups. In particular, there was a concern about an overrepresentation of participants in the retail dispensing group who were able and willing to pay the co‐payment. This subgroup might systematically differ from those selecting the cost‐free courier delivery option, in socioeconomic status, and other factors that may influence viral suppression.

### 2.3 Outcome and measures

The primary outcome of the study was viral suppression, defined as a VL <400 copies per ml. Participants contributed multiple VL measurements to the analysis, usually around two per person‐year of follow‐up, taken during the study period and after ART initiation. Measurements taken less than 5 months after a previous measurement were excluded to avoid double counting of repeated VL tests taken to confirm virologic treatment failure.

At each VL measurement, we identified the ART distribution method (courier delivery or retail dispensing) and ART regimen by carrying forward information from the most recent ART claim submitted before the VL measurement. ART regimens were classified into three groups based on their constituent drugs: one integrase inhibitor and two nucleoside reverse transcriptase inhibitors (II+2NRTI), one protease inhibitor and two nucleoside reverse transcriptase inhibitors (PI+2NRTI), and one non‐nucleoside reverse transcriptase inhibitors and two nucleoside reverse transcriptase inhibitors (NNRTI+2NRTI).

We categorized the medical insurance schemes into three groups, namely: separate categories for the two largest schemes “A” and “B,” and a third “Other” category consisting of the several remaining, smaller schemes. Scheme B introduced co‐payments for retail dispensing and was thus excluded from the analysis. Age was grouped into six categories: 15−29, 30−39, 40−49, 50−59, 60−69 and 70+ years. Sex was recorded as male or female. The period of study was divided into calendar years: 2011−2013, 2014−2016, 2017−2019 and 2020−2022. We classified members as having a history of mental health diagnoses if they received an International Classification of Diseases 10th Edition (ICD‐10) code in the range of F00−F99, with the exception of the F17 code, which is related to smoking use disorder [21]. These diagnoses were recorded by healthcare providers during routine care.

### 2.4 Statistical analysis

We used descriptive statistics to examine individual‐level characteristics and the distribution of predictors of viral suppression at the time of each VL test, stratified by ART distribution method (courier delivery or retail dispensing). We tested for significant differences (*p*‐value <0.05) between characteristics in the courier delivery and retail dispensing groups using chi‐squared tests (binary and categorical variables) and rank sum tests (continuous variables). We evaluated the percentage of individuals on courier delivery by medical insurance scheme (A or Other) and by month of the study period.

Using logistic regression, we calculated unadjusted and adjusted odds ratios (ORs) for associations between ART distribution method (courier delivery vs. retail dispensing) and viral suppression. In the adjusted analyses, we controlled for the following variables measured at VL testing: age, sex, calendar year, ART regimen, history of mental health diagnosis and medical insurance scheme (A vs. other). Furthermore, we computed unadjusted and adjusted ORs for each of the periods 2011−2013, 2014−2016, 2017−2019 and 2020−2022. We report crude percentages and adjusted mean percentages of viral suppression in participants on courier delivery or retail dispensing for each period and overall. The adjusted mean percentages were derived from the logistic regression models. We used generalized estimating equations with exchangeable correlation structure to account for repeated VL testing of the same individual in all of the above regression models [22, 23]. We repeated these analyses separately for medical schemes A and Other and by sex. In the analysis of scheme A, we left‐truncated follow‐up at the start of 2016 due to the limited numbers of participants in the scheme before that year. We performed sensitivity analyses using VL suppression thresholds of 50 and 1000 copies per ml, and analyses where we lagged the ART distribution method by 6 or 12 months.

We computed unadjusted and adjusted hazard ratios (HRs) for factors associated with an increased risk of changing ART distribution method from courier delivery to retail dispensing and from retail dispensing to courier delivery, using a two‐state model and proportional hazards Cox regression [24, 25]. Models included the variables controlled for in analyses above. We coded age, calendar year, history of mental illness and ART regimen as time‐updated variables for these analyses. Additionally, we included a time‐updated binary variable indicating whether the individual was virally suppressed. Statistical analyses were performed with R 4.2.3 (R Foundation for Statistical Computing, Vienna, Austria).

### 2.5 Ethical considerations

AfA received ethical approval from the Human Research Ethics Committee (HREC) of the University of Cape Town, South Africa to contribute data to the International epidemiology Databases to Evaluate AIDS‐Southern Africa collaboration (IeDEA‐SA) [26]. HREC and the Cantonal Ethics Committee Bern, Switzerland authorized the analysis of the database. Beneficiaries of the medical insurance schemes or their guardians provided consent for their data to be used in research.

## 3 RESULTS

We included 68,720 individuals in our analysis, with 58,372 individuals from scheme B having been excluded. Among participants included in the analysis, the median age at the first VL test was 40 years (IQR 34−47) and 39,406 (57.3%) were female (Table 1). The majority of participants (87.3%) were receiving a regimen containing one NNRTI and two NRTIs at their first VL test, and by the end of follow‐up, over half (52.8%) had received a mental health diagnosis (Table 1).

**Table 1:** Characteristics of participants included in the main analysis, stratified by ART distribution method at first viral load test

	ART distribution method at first viral load test		
	Retail	Courier	Total
**Number of individuals**	38,060	30,660	68,720
**Ever mental health diagnosis** ^a^ **, *n* (%)**			
No	16,909 (44.4)	15,551 (50.7)	32,460 (47.2)
Yes	21,151 (55.6)	15,109 (49.3)	36,260 (52.8)
**Sex, *n* (%)**			
Male	16,461 (43.3)	12,853 (41.9)	29,314 (42.7)
Female	21,599 (56.7)	17,807 (58.1)	39,406 (57.3)
**Age at first viral load test, *n* (%)**			
15−29 years	4602 (12.1)	2710 (8.8)	7312 (10.6)
30−39 years	15,615 (41.0)	11,194 (36.5)	26,809 (39.0)
40−49 years	12,053 (31.7)	11,186 (36.5)	23,239 (33.8)
50−59 years	5128 (13.5)	4976 (16.2)	10,104 (14.7)
60−69 years	590 (1.6)	539 (1.8)	1129 (1.6)
70+ years	72 (0.2)	55 (0.2)	127 (0.2)
Median [IQR]	39.28 [33.74, 46.31]	41.09 [35.32, 47.85]	40.08 [34.41, 47.09]
**Year at first viral load test, *n* (%)**			
2011−2013	5844 (15.4)	7396 (24.1)	13,240 (19.3)
2014−2016	15,969 (42.0)	12,950 (42.2)	28,919 (42.1)
2017−2019	11,540 (30.3)	7673 (25.0)	19,213 (28.0)
2020−2022	4707 (12.4)	2641 (8.6)	7348 (10.7)
**ART regimen at first viral load test, *n* (%)**			
NNRTI+2NRTI	33,276 (87.4)	26,683 (87.0)	59,959 (87.3)
II+2NRTI	1211 (3.2)	589 (1.9)	1800 (2.6)
PI+2NRTI	3573 (9.4)	3388 (11.1)	6961 (10.1)
**Ever switched distribution method** ^b^ **, *n* (%)**			
No	32,502 (85.4)	24,120 (78.7)	56,622 (82.4)
Yes	5558 (14.6)	6540 (21.3)	12,098 (17.6)

Abbreviations: ART, antiretroviral therapy; II, integrase inhibitor; IQR, inter‐quartile range; NNRTI, non‐nucleoside reverse transcriptase inhibitor; NRTI, nucleoside reverse transcriptase inhibitor; PI, protease inhibitor.

^a^ History of mental illness at the time of the last viral load test.

^b^ Changed at least once from retail to courier or courier to retail at the time of successive viral load measurements.

The participants included in the analysis contributed 442,619 VL measurements to the study (Table 2). The median number of VL tests per individual was 6 (IQR 3−10), the median number of days between a person's first and last VL test was 1289 (IQR 391−2015), and the median number of days between successive VL tests within individuals was 194 (IQR 180−239). Fewer than half of the VL tests (185,701, 42.0%) were taken while the participant was receiving ART via courier delivery (Table 2). Participants who received ART via courier delivery or retail dispensing were broadly similar in demographics and clinical characteristics, albeit with statistically significant differences (*p*<0.001 for all factors). Minor differences were observed, such as a slightly lower proportion of individuals with a mental health diagnosis, a marginally greater percentage of females in the courier than in the retail group. The median age was also marginally higher in the courier group (43.65 vs. 42.33 in retail, Table 2). We observed a higher percentage of people switching distribution methods among those starting in the courier group compared with those starting in the retail group. A total of 398,901 (90.1%) VL measurements were <400 copies per ml, with a similar proportion of tests indicating viral suppression among participants receiving ART by courier delivery (90.4%) and those receiving ART by retail dispensing (90.0%).

**Table 2:** Characteristics of participants at each viral load test, stratified by ART distribution method at time of viral load testing

	ART distribution method		
	Retail	Courier	Total
**Number of viral load tests**	256,918	185,701	442,619
**History of mental health diagnosis, *n* (%)**			
No	125,215 (48.7)	97,458 (52.5)	222,673 (50.3)
Yes	131,703 (51.3)	88,243 (47.5)	219,946 (49.7)
**Sex, *n* (%)**			
Male	111,627 (43.4)	79,274 (42.7)	190,901 (43.1)
Female	145,291 (56.6)	106,427 (57.3)	251,718 (56.9)
**Age, *n* (%)**			
15−29 years	15,657 (6.1)	9185 (4.9)	24,842 (5.6)
30−39 years	85,403 (33.2)	54,526 (29.4)	139,929 (31.6)
40−49 years	97,433 (37.9)	72,470 (39.0)	169,903 (38.4)
50−59 years	49,734 (19.4)	42,363 (22.8)	92,097 (20.8)
60−69 years	7680 (3.0)	6360 (3.4)	14,040 (3.2)
70+ years	1011 (0.4)	797 (0.4)	1808 (0.4)
Median [IQR]	42.33 [36.75, 49.24]	43.65 [37.79, 50.49]	42.87 [37.18, 49.81]
**Year of viral load test, *n* (%)**			
2011−2013	17,322 (6.7)	18,435 (9.9)	35,757 (8.1)
2014−2016	44,441 (17.3)	29,725 (16.0)	74,166 (16.8)
2017−2019	111,761 (43.5)	81,371 (43.8)	193,132 (43.6)
2020−2022	83,394 (32.5)	56,170 (30.2)	139,564 (31.5)
**ART regimen at viral load test, *n* (%)**			
NNRTI+2NRTI	215,377 (83.8)	156,727 (84.4)	372,104 (84.1)
II+2NRTI	6864 (2.7)	3758 (2.0)	10,622 (2.4)
PI+2NRTI	34,677 (13.5)	25,216 (13.6)	59,893 (13.5)
**Viral suppression, *n* (%)**			
<400 copies/ml	231,111 (90.0)	167,790 (90.4)	398,901 (90.1)
≥400 copies/ml	25,807 (10.0)	17,911 (9.6)	43,718 (9.9)

*Note*: Participants could contribute multiple viral load tests.

Abbreviations: ART, antiretroviral therapy; II, integrase inhibitor; IQR, inter‐quartile range; NNRTI, non‐nucleoside reverse transcriptase inhibitor; NRTI, nucleoside reverse transcriptase inhibitor; PI, protease inhibitor.

**Figure 1:**
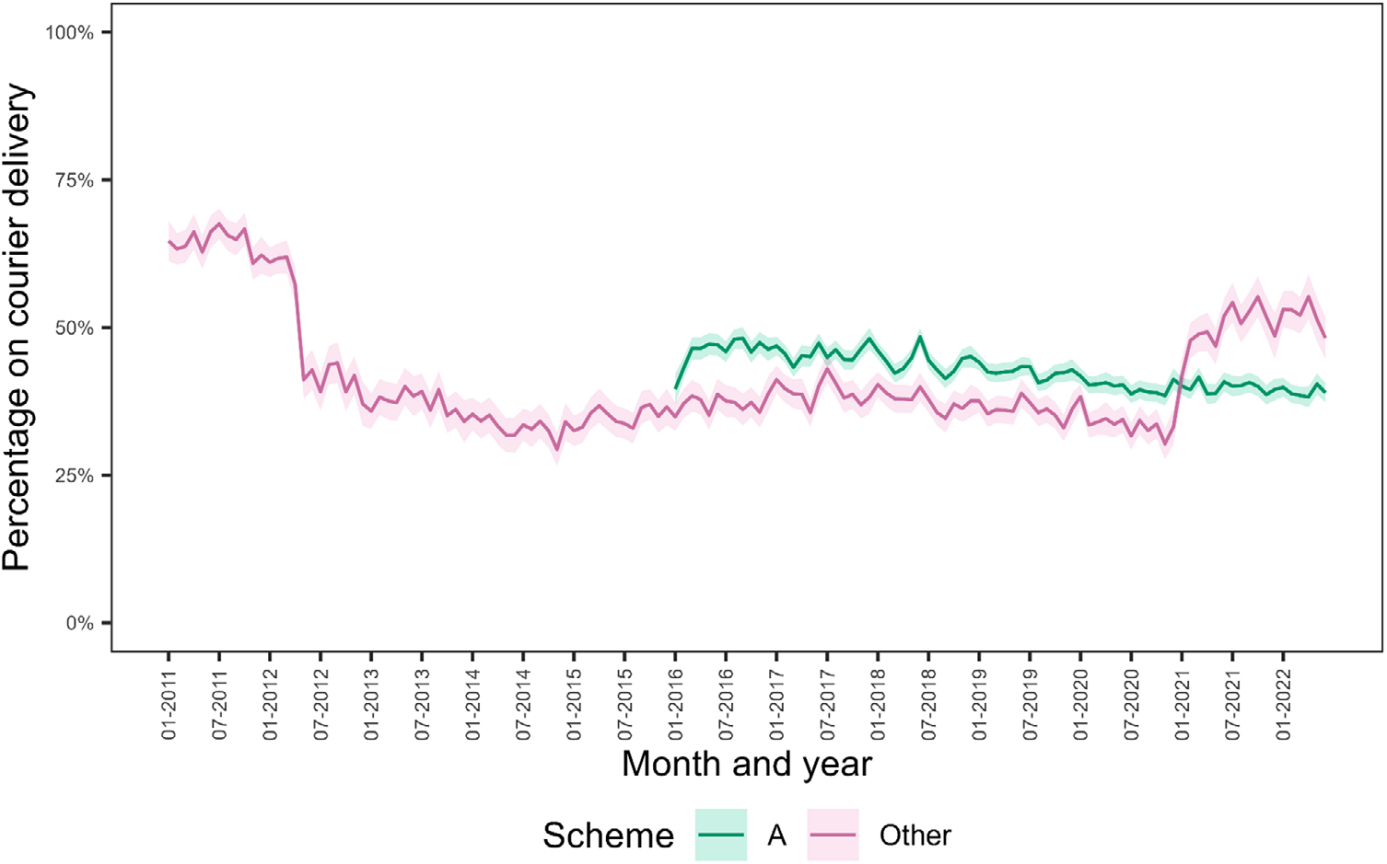
Percentage of individuals receiving antiretroviral therapy via courier delivery, by month and by medical scheme. The shaded areas represent 95% confidence intervals. Percentages for scheme A are not shown before 2016 due to the limited numbers of individuals in the scheme during that period.

Figure 1 shows the monthly percentage of people receiving ART by courier delivery by medical insurance scheme. Courier delivery use in scheme A remained between 38% and 49% from the start of 2016 to the end of the study period. In the schemes from the “Other” category, the percentage of individuals on courier delivery was 64.7% (95% CI 61.3−68.1) at the start of the study period, 35.3% (95% CI 32.9−37.7) at the start of 2016 and 49.2% (95% CI 45.6−52.7) at the end of the study period. The COVID‐19 pandemic had no impact on the proportion of individuals employing courier ART delivery.

Table 3 shows unadjusted and adjusted ORs for viral suppression comparing the two ART distribution methods, by calendar period and overall. After adjusting for history of mental illness, sex, age, ART regimen, calendar year and medical scheme, courier ART delivery was associated with 5% higher odds of viral suppression (adjusted OR 1.05, 95% CI 1.02−1.08) compared to retail dispensing. The strength and direction of this association varied by calendar period, from an adjusted OR of 1.37 (95% CI 1.27−1.48) in 2011−2013, to an adjusted OR of 0.95 (95% CI 0.91−0.99) in 2017−2019, and to an adjusted OR of 1.02 (95% CI 0.97−1.07) in 2020−2022 (Table 3). The adjusted ORs for other factors are shown in Table S1. Female sex, older age and more recent tests were associated with higher odds of viral suppression, while having a history of mental illness was associated with lower odds of viral suppression. The adjusted mean percentage of viral suppression increased from the 2011−2013 period to the 2020−2022 period, both among people on retail ART dispensing (80.3%−91.7%) and among people receiving ART via courier delivery (84.8%−91.8%, Table 3). The strength of the association between ART distribution method and viral suppression varied by medical insurance scheme (Tables S2 and S3) and by sex (Tables S4 and S5 ). Lagging the delivery method by 6 or 12 months reduced this association (Tables S6 and S7), while changing the viral suppression threshold to 50 or 1000 copies per ml did not change it (Tables S8 and S9).

**Table 3:** Unadjusted and adjusted odds ratios for viral suppression (<400 RNA viral load copies per ml), comparing individuals on courier ART delivery to those on retail ART dispensing, and crude and adjusted mean probabilities of viral suppression for both ART distribution methods

	Calendar period				
	2011−2013	2014−2016	2017−2019	2020−2022	Overall
**Unadjusted OR for viral suppression**					
Retail dispensing	1	1	1	1	1
Courier delivery	1.48 (1.37−1.59)	1.14 (1.07−1.21)	0.97 (0.93−1.01)	1.03 (0.98−1.08)	1.02 (0.99−1.05)
**Adjusted OR for viral suppression** ^a^					
Retail dispensing	1	1	1	1	1
Courier delivery	1.37 (1.27−1.48)	1.06 (1.00−1.13)	0.95 (0.91−0.99)	1.02 (0.97−1.07)	1.05 (1.02−1.08)
**Crude percentage virally suppressed**					
Retail dispensing	79.3 (78.4−80.1)	87.8 (87.4−88.2)	88.7 (88.4−89.0)	90.6 (90.3−90.8)	87.6 (87.3−87.8)
Courier delivery	85.0 (84.3−85.6)	89.1 (88.6−89.5)	88.4 (88.0−88.7)	90.8 (90.5−91.1)	87.8 (87.5−88.1)
**Adjusted mean percentage virally suppressed** ^a^					
Retail dispensing	80.3 (74.3−85.1)	88.6 (87.2−89.9)	92.4 (91.1−93.6)	91.7 (90.9−92.4)	90.5 (90.0−91.1)
Courier delivery	84.8 (79.9−88.7)	89.2 (87.9−90.5)	92.1 (90.6−93.3)	91.8 (91.0−92.5)	90.9 (90.4−91.5)

*Note*: Participants could contribute multiple viral load tests. Generalized estimating equations were used to account for repeated viral load measurements from the same individual. Results are provided by calendar period and overall.

Abbreviations: ART, antiretroviral therapy; OR, odds ratio.

^a^ Adjusted for history of mental illness, sex, age, ART regimen, calendar year (overall analysis only) and medical scheme.

Individuals who were virally suppressed (<400 copies per ml) were less likely to switch from retail dispensing to courier delivery (adjusted HR 0.77, 95% CI 0.74−0.81) and from courier delivery to retail dispensing (adjusted HR 0.66, 95% 0.63−0.69) compared to unsuppressed individuals (Table 4). Individuals with a history of mental illness were more likely to switch from courier delivery to retail dispensing than individuals without a mental illness (adjusted HR 1.19, 95% CI 1.15−1.22). Compared to men, women were more likely to switch from retail dispensing to courier delivery (adjusted HR 1.18, 95% CI 1.14−1.21) and from courier delivery to retail dispensing (adjusted HR 1.06, 95% CI 1.02−1.09). Rates of switching ART distribution method increased significantly over the study period.

**Table 4:** Unadjusted and adjusted hazard ratios for factors associated with a switch of ART distribution method (courier to retail or retail to courier)

	Retail to courier		Courier to retail	
	Unadjusted	Adjusted	Unadjusted	Adjusted
**Viral suppression (<400 copies per ml)**				
No	1	1	1	1
Yes	0.76 (0.73−0.79)	0.77 (0.74−0.81)	0.64 (0.62−0.67)	0.66 (0.63−0.69)
**History of mental illness**				
No	1	1	1	1
Yes	1.02 (0.99−1.05)	0.99 (0.96−1.02)	1.23 (1.19−1.27)	1.19 (1.15−1.22)
**Sex**				
Male	1	1	1	1
Female	1.08 (1.05−1.12)	1.18 (1.14−1.21)	1.02 (0.99−1.04)	1.06 (1.02−1.09)
**Age**				
15−29	1	1	1	1
30−39	0.96 (0.90−1.03)	1.03 (0.97−1.10)	0.88 (0.82−0.94)	0.94 (0.88−1.01)
40−49	1.08 (1.02−1.16)	1.19 (1.11−1.27)	0.86 (0.81−0.92)	0.94 (0.88−1.01)
50−59	1.30 (1.21−1.39)	1.47 (1.37−1.58)	0.88 (0.83−0.95)	0.97 (0.90−1.04)
60−69	1.34 (1.22−1.48)	1.54 (1.40−1.71)	0.88 (0.80−0.98)	0.98 (0.89−1.09)
70+	1.42 (1.15−1.76)	1.65 (1.33−2.04)	0.83 (0.66−1.05)	0.94 (0.74−1.19)
**Calendar year**				
2011−2013	1	1	1	1
2014−2016	0.88 (0.82−0.95)	0.92 (0.85−0.99)	2.20 (2.03−2.39)	2.21 (2.04−2.40)
2017−2019	1.07 (1.00−1.15)	1.12 (1.05−1.20)	2.46 (2.27−2.66)	2.44 (2.25−2.64)
2020−2022	1.43 (1.33−1.53)	1.44 (1.34−1.55)	2.77 (2.55−3.01)	2.64 (2.43−2.87)
**ART regimen**				
NNRTI+2NRTI	1	1	1	1
II+2NRTI	1.79 (1.66−1.91)	1.53 (1.42−1.65)	1.86 (1.72−2.01)	1.67 (1.55−1.80)
PI+2NRTI	1.27 (1.22−1.32)	1.22 (1.17−1.27)	1.19 (1.15−1.24)	1.14 (1.10−1.19)

*Note*: The adjusted models include all factors listed in the table. The hazards were stratified by medical insurance scheme (A, Other) in both the unadjusted and adjusted models.

Abbreviations: ART, antiretroviral therapy; II, integrase inhibitor; NNRTI, non‐nucleoside reverse transcriptase inhibitor; NRTI, nucleoside reverse transcriptase inhibitor; PI, protease inhibitor.

## 4 DISCUSSION

Our study investigated the association between courier delivery of ART and viral suppression among people enrolled in the AfA programme in South Africa between 2011 and 2022. To our knowledge, this is the largest study to date to assess the impact of courier ART delivery on viral suppression rates. Individuals receiving ART by courier delivery had 5% higher odds of achieving viral suppression (<400 RNA VL copies/ml) compared to individuals receiving ART through traditional retail dispensing, independent of known and measured risk factors for viral non‐suppression. However, the association between courier ART delivery and viral suppression varied by calendar period, with the strongest association at the start of the study period (2011−2013), and no significant association seen in the most recent period (2020−2022). The proportion of VL tests indicating viral suppression was high (>90% by the end of the study period) in both the courier delivery and retail dispensing groups. Individuals who were virally suppressed were less likely to change their dispensing method (courier delivery to retail pharmacy or vice versa) than those who were not.

Several studies have assessed the association between courier ART delivery and ART outcomes. Our findings from the early stages of our study period corroborate the results of a previous cohort study of the same programme between the years 2002 and 2011, which found that courier delivery of ART was associated with significant improvements in CD4 counts, higher viral suppression rates and lower mortality compared to retail ART dispensing [14]. Our findings from the later stages of our study period are consistent with the results of a randomized trial in Tanzanian healthcare facilities that found community delivery of ART led to non‐inferior virological outcomes compared to standard of care [15]. Smaller retrospective cohort studies from the United States and the United Kingdom have shown mixed results. A study from the United States found that sustained viral suppression among low‐income individuals using delivery service pharmacies was consistently greater compared to individuals using in‐store pharmacy [16]. A study from an inner London HIV treatment centre similarly found home delivery of ART to be associated with a lower risk of virological failure compared with standard clinic pharmacy dispensing of medication [17]. In contrast, another London‐based study found no significant change in the HIV VL, CD4% and adherence status as a result of change in the mode of supply from usual care to home delivery of ART [18]. A single‐centre US‐based study found no difference in viral suppression rates with mail order pharmacy versus in‐person pharmacy collection [19].

There are several potential explanations for the higher rates of viral suppression associated with courier delivery in the early stages of our study. Firstly, early adopters of courier pharmacies may have been individuals who would have benefited most from the convenience of having their ART delivered to their homes. As courier pharmacies became more established, more people may have opted to use the service, including those for whom it made little difference whether they had their ART delivered or whether they collected it from the pharmacy themselves. Early adopters of courier pharmacies may also have been more motivated and engaged in their care, contributing to their comparatively better treatment outcomes. Secondly, healthcare providers may have offered courier pharmacy delivery to non‐suppressed patients in later years to improve their adherence. As a result, individuals who were struggling with adherence may have been more likely to receive courier delivery of ART, which could have contributed to the lower observed benefit in later years. However, this hypothesis is not supported by the results from our analysis of factors associated with switching from retail pharmacy to courier delivery. Groups with lower adherence, for example individuals with mental health conditions, males, adolescents and young adults, were less likely to switch from retail to courier compared to those groups known for better adherence, such as individuals without mental health conditions, females and older adults. Finally, other unmeasured confounders may have played a role in the observed trends.

Our study has several limitations. First, we determined the type of ART distribution (courier delivery or retail dispensing) associated with each VL measurement by using the distribution method from the most recent ART claim submitted before the VL measurement. This approach may have resulted in carry‐over between the two groups if individuals switched from one distribution method to another during the study period. Second, our study population consisted of individuals who were enrolled in a private‐sector ART programme, which may restrict the generalizability of our findings to other healthcare settings or populations. Third, our study was observational and cannot establish causality between ART distribution type and viral suppression. Randomized studies would be needed to measure the effect of courier services accurately. Fourth, our study did not include individuals with no VL tests.

Courier delivery of ART may be of benefit in ways not demonstrated by this study. The convenience of receiving medications at home or at work may reduce the burden of HIV treatment on individuals, especially for those who have long travel or waiting times or transport costs associated with drug refills [5, 6]. Additionally, courier delivery of medication can help protect the privacy of individuals. Finally, courier services may be especially useful when movement and transportation are restricted, for example, during lockdown measures and extreme weather conditions, making it difficult for individuals to access their HIV treatment [12, 13, 27].

## 5 CONCLUSIONS

Data on the impact of ART courier delivery on HIV treatment outcomes are scarce. Our study suggests that courier delivery of ART is a viable alternative to traditional retail dispensing, as it was associated with higher viral suppression until 2016 and similar suppression rates in recent years. Courier delivery of ART may provide additional benefits for individuals who have difficulty accessing treatment. Further research is needed to investigate the potential benefits and drawbacks of courier delivery of ART in both the public and private healthcare sectors. Programmatic and economic factors associated with courier services also warrant further exploration.

## COMPETING INTERESTS

NF, MH and AMN Namubiru are employed by Medscheme, the company that facilitated the provision of the data for this study.  LD is employed by AfA. Both Medscheme and AfA fall under the AfroCentric Group, a company providing services and products to the healthcare sector, including a courier pharmacy company.

## AUTHORS’ CONTRIBUTIONS

ME and AH obtained funding for the study. YR and AH wrote the first draft of the study protocol, which was revised by all authors. YR performed the analyses. YR, NF, and AH wrote the first draft of the paper. All other authors contributed to the interpretation of results and critically revised the manuscript for intellectual content. All authors have approved the final version of the manuscript for publication.

## FUNDING

Research reported in this study was supported by the U.S. National Institutes of Health's National Institute of Allergy and Infectious Diseases, the Eunice Kennedy Shriver National Institute of Child Health and Human Development, Division of Cancer Epidemiology and Genetics, National Cancer Institute, the National Institute of Mental Health, the National Institute on Drug Abuse, the National Heart, Lung, and Blood Institute, the National Institute on Alcohol Abuse and Alcoholism, the National Institute of Diabetes and Digestive and Kidney Diseases and the Fogarty International Center under Award Number U01AI069924. ADH and ME were supported by the Swiss National Science Foundation under Award Numbers 193381 and 189498.

## DISCLAIMER

The content is solely the responsibility of the authors and does not necessarily represent the official views of the National Institutes of Health.

## Supporting information




**Supplemental Table 1**: Adjusted odds ratios for viral suppression (<400 RNA viral load per mL) for various factors. The models are adjusted for the variables listed in the table, as well as for the ART distribution method (courier or retail). Results are provided by calendar period and overall.
**Supplemental Table 2**. Unadjusted and adjusted odds ratios for viral suppression (<400 RNA viral load copies per mL) in medical insurance scheme A, comparing individuals on courier ART delivery to those on retail ART dispensing, and crude and adjusted mean probabilities of viral suppression for both ART distribution methods. Results are provided by calendar period and overall. Data are left‐truncated at the start of 2016.
**Supplemental Table 3**: Unadjusted and adjusted odds ratios for viral suppression (<400 RNA viral load copies per mL) in medical insurance schemes other than A and B, comparing individuals on courier ART delivery to those on retail ART dispensing, and crude and adjusted mean probabilities of viral suppression for both ART distribution methods. Results are provided by calendar period and overall.
**Supplemental Table 4**: Unadjusted and adjusted odds ratios for viral suppression (<400 RNA viral load copies per mL), comparing men on courier ART delivery to those on retail ART dispensing, and crude and adjusted mean probabilities of viral suppression for both ART distribution methods. Results are provided by calendar period and overall.
**Supplemental Table 5**: Unadjusted and adjusted odds ratios for viral suppression (<400 RNA viral load copies per mL), comparing women on courier ART delivery to those on retail ART dispensing, and crude and adjusted mean probabilities of viral suppression for both ART distribution methods. Results are provided by calendar period and overall.
**Supplemental Table 6**: Unadjusted and adjusted odds ratios for viral suppression (<400 RNA viral load copies per mL), comparing individuals on courier ART delivery to those on retail ART dispensing, and crude and adjusted mean probabilities of viral suppression for both ART distribution methods. Results are provided by calendar period and overall. The ART delivery type variable has been lagged by 6 months.
**Supplemental Table 7**: Unadjusted and adjusted odds ratios for viral suppression (<400 RNA viral load copies per mL), comparing individuals on courier ART delivery to those on retail ART dispensing, and crude and adjusted mean probabilities of viral suppression for both ART distribution methods. Results are provided by calendar period and overall. The delivery type variable has been lagged by 12 months.
**Supplemental Table 8**: Unadjusted and adjusted odds ratios for viral suppression (<50 RNA viral load copies per mL), comparing individuals on courier ART delivery to those on retail ART dispensing, and crude and adjusted mean probabilities of viral suppression for both ART distribution methods. Results are provided by calendar period and overall.
**Supplemental Table 9**: Unadjusted and adjusted odds ratios for viral suppression (<1000 RNA viral load copies per mL), comparing individuals on courier ART delivery to those on retail ART dispensing, and crude and adjusted mean probabilities of viral suppression for both ART distribution methods. Results are provided by calendar period and overall.

## Data Availability

Data were obtained from the International epidemiology Databases to Evaluate AIDS‐Southern Africa (IeDEA‐SA). Due to legal and ethical restrictions, the data cannot be made publicly available. For inquiries about the data, readers can contact IeDEA‐SA through the online form available at https://www.iedea‐sa.org/contact‐us/.
