# Supplementary material for: Courier delivery of antiretroviral therapy: a cohort study of a South African private‐sector HIV programme
Source: J Int AIDS Soc. 2024 Sep 18;27(9):e26360. doi: 10.1002/jia2.26360 (PMC11410889; doi:10.1002/jia2.26360)
Supplement: Supplementary file 1 — Supplemental Table 1: Adjusted odds ratios for viral suppression (<400 RNA viral load per mL) for various factors. The models are adjusted for the variables listed in the table, as well as for the ART distribution method (courier or retail). Results are provided by calendar period and overall. Supplemental Table 2. Unadjusted and adjusted odds ratios for viral suppression (<400 RNA viral load copies per mL) in medical insurance scheme A, comparing individuals on courier ART delivery to those on retail ART dispensing, and crude and adjusted mean probabilities of viral suppression for both ART distribution methods. Results are provided by calendar period and overall. Data are left‐truncated at the start of 2016. Supplemental Table 3: Unadjusted and adjusted odds ratios for viral suppression (<400 RNA viral load copies per mL) in medical insurance schemes other than A and B, comparing individuals on courier ART delivery to those on retail ART dispensing, and crude and adjusted mean probabilities of viral suppression for both ART distribution methods. Results are provided by calendar period and overall. Supplemental Table 4: Unadjusted and adjusted odds ratios for viral suppression (<400 RNA viral load copies per mL), comparing men on courier ART delivery to those on retail ART dispensing, and crude and adjusted mean probabilities of viral suppression for both ART distribution methods. Results are provided by calendar period and overall. Supplemental Table 5: Unadjusted and adjusted odds ratios for viral suppression (<400 RNA viral load copies per mL), comparing women on courier ART delivery to those on retail ART dispensing, and crude and adjusted mean probabilities of viral suppression for both ART distribution methods. Results are provided by calendar period and overall. Supplemental Table 6: Unadjusted and adjusted odds ratios for viral suppression (<400 RNA viral load copies per mL), comparing individuals on courier ART delivery to those on retail ART dispensing, a [file JIA2-27-e26360-s001.docx]

**Supplemental Table 1: Adjusted odds ratios for viral suppression (<400 RNA viral load per mL) for various factors. The models are adjusted for the variables listed in the table, as well as for the ART distribution method (courier or retail). Results are provided by calendar period and overall.**

|  |  | **Calendar period** | | | | **Overall** |
| --- | --- | --- | --- | --- | --- | --- |
|  |  | 2011-2013  (95% CI) | 2014-2016  (95% CI) | 2017-2019 | 2020-2022 |  |
| **History of mental health diagnosis at viral load test** |  |  |  |  |  |  |
| No |  | 1 | 1 | 1 | 1 | 1 |
| Yes |  | 0.98 (0.89-1.06) | 0.96 (0.90-1.01) | 0.90 (0.87-0.94) | 0.89 (0.84-0.94) | 0.94 (0.91-0.96) |
| **Sex** |  |  |  |  |  |  |
| Male |  | 1 | 1 | 1 | 1 | 1 |
| Female |  | 1.41 (1.30-1.53) | 1.45 (1.37-1.55) | 1.58 (1.51-1.66) | 1.66 (1.57-1.75) | 1.54 (1.49-1.60) |
| **Age at viral load test** |  |  |  |  |  |  |
| 15-29 years |  | 0.50 (0.44-0.57) | 0.47 (0.42-0.52) | 0.50 (0.46-0.53) | 0.41 (0.37-0.45) | 0.57 (0.54-0.60) |
| 30-39 years |  | 1 | 1 | 1 | 1 | 1 |
| 40-49 years |  | 0.76 (0.71-0.83) | 0.78 (0.73-0.83) | 0.81 (0.77-0.85) | 0.81 (0.76-0.86) | 0.86 (0.83-0.88) |
| 50-59 years |  | 1.13 (1.02-1.25) | 1.03 (0.95-1.12) | 1.09 (1.04-1.15) | 1.17 (1.10-1.25) | 1.06 (1.02-1.09) |
| 60-69 years |  | 1.42 (1.11-1.81) | 1.53 (1.24-1.90) | 1.52 (1.33-1.73) | 1.65 (1.43-1.90) | 1.26 (1.17-1.35) |
| 70+ years |  | 2.08 (0.97-4.50) | 3.09 (1.50-6.38) | 1.89 (1.23-2.89) | 3.09 (1.87-5.10) | 1.60 (1.33-1.91) |
| **ART regimen at viral load test** |  |  |  |  |  |  |
| NNRTI+2NRTI |  | 1 | 1 | 1 | 1 | 1 |
| II+2NRTI* |  | 1.00 (0.87-1.14) | 0.59 (0.53-0.65) | 3.04 (1.88-4.90) | 1.41 (1.26-1.57) | 2.53 (2.21-2.89) |
| PI+2NRTI* |  |  |  | 0.75 (0.68-0.83) | 0.37 (0.34-0.40) | 1.17 (1.07-1.28) |
| **Medical scheme** |  |  |  |  |  |  |
| A |  | 1 | 1 | 1 | 1 | 1 |
| Other |  | 1.24 (0.68-2.27) | 0.63 (0.59-0.67) | 0.88 (0.84-0.93) | 0.94 (0.89-1.00) | 0.84 (0.80-0.87) |
| **Year of viral load test, n (%)** |  |  |  |  |  |  |
| 2011-2013 |  |  |  |  |  | 1 |
| 2014-2016 |  |  |  |  |  | 1.33 (1.28-1.38) |
| 2017-2019 |  |  |  |  |  | 1.40 (1.34-1.46) |
| 2020-2022 |  |  |  |  |  | 1.52 (1.46-1.59) |

Participants could contribute multiple viral load tests. Generalized estimating equations were used to account for repeated viral load measurements from the same individual. Abbreviations: ART, antiretroviral therapy; NNRTI, non-nucleoside reverse transcriptase inhibitors; NRTI, nucleoside reverse transcriptase inhibitors; II, integrase inhibitor; PI, protease inhibitor. *II+2RNTI grouped with PI+NRTI for the 2011-2016 period.

Supplemental Table 2. Unadjusted and adjusted odds ratios for viral suppression (<400 RNA viral load copies per mL) in medical insurance scheme A, comparing individuals on courier ART delivery to those on retail ART dispensing, and crude and adjusted mean probabilities of viral suppression for both ART distribution methods. Results are provided by calendar period and overall. Data are left-truncated at the start of 2016.

|  | **Calendar period** | | | **Overall** |
| --- | --- | --- | --- | --- |
|  | 2016-2017 | 2018-2019 | 2020-2022 |  |
| **Unadjusted OR for viral suppression** |  |  |  |  |
| Retail dispensing | 1 | 1 | 1 | 1 |
| Courier delivery | 0.98 (0.92-1.04) | 0.95 (0.90-1.01) | 0.98 (0.93-1.04) | 0.96 (0.92-1.00) |
| **Adjusted OR for viral suppression*** |  |  |  |  |
| Retail dispensing | 1 | 1 | 1 | 1 |
| Courier delivery | 0.97 (0.92-1.04) | 0.95 (0.89-1.00) | 0.98 (0.93-1.04) | 0.98 (0.94-1.02) |
| **Crude percentage virally suppressed** |  |  |  |  |
| Retail dispensing | 89.3 (88.9-89.7) | 89.4 (89.1-89.8) | 90.7 (90.3-91.0) | 89.1 (88.8-89.4) |
| Courier delivery | 89.1 (88.7-89.6) | 88.9 (88.5-89.4) | 90.5 (90.1-90.9) | 88.6 (88.3-89.0) |
| **Adjusted mean percentage virally suppressed*** |  |  |  |  |
| Retail dispensing | 91.8 (86.8-95.0) | 91.5 (89.9-92.9) | 91.7 (90.8-92.6) | 92.4 (91.7-93.0) |
| Courier delivery | 91.6 (86.5-94.9) | 91.1 (89.3-92.5) | 91.6 (90.6-92.5) | 92.3 (91.6-92.9) |

Participants could contribute multiple viral load tests. Generalized estimating equations were used to account for repeated viral load measurements from the same individual. Abbreviations: ART, antiretroviral therapy; OR, odds ratio; *Adjusted for history of mental illness, sex, age, ART regimen, and calendar year (overall analysis only).

Supplemental Table 3: Unadjusted and adjusted odds ratios for viral suppression (<400 RNA viral load copies per mL) in medical insurance schemes other than A and B, comparing individuals on courier ART delivery to those on retail ART dispensing, and crude and adjusted mean probabilities of viral suppression for both ART distribution methods. Results are provided by calendar period and overall.

|  | **Calendar period** | | | | **Overall** |
| --- | --- | --- | --- | --- | --- |
|  | 2011-2013 | 2014-2016 | 2017-2019 | 2020-2022 |  |
| **Unadjusted OR for viral suppression** |  |  |  |  |  |
| Retail dispensing | 1 | 1 | 1 | 1 | 1 |
| Courier delivery | 1.49 (1.38-1.60) | 1.16 (1.07-1.26) | 1.01 (0.94-1.09) | 1.14 (1.05-1.25) | 1.11 (1.07-1.16) |
| **Adjusted OR for viral suppression*** |  |  |  |  |  |
| Retail dispensing | 1 | 1 | 1 | 1 | 1 |
| Courier delivery | 1.38 (1.28-1.49) | 1.11 (1.02-1.21) | 0.98 (0.91-1.05) | 1.11 (1.01-1.22) | 1.14 (1.09-1.19) |
| **Crude percentage virally suppressed** |  |  |  |  |  |
| Retail dispensing | 79.3 (78.4-80.2) | 85.0 (84.3-85.6) | 88.1 (87.6-88.6) | 90.4 (89.8-90.9) | 85.4 (85.0-85.8) |
| Courier delivery | 85.0 (84.3-85.7) | 86.8 (86.0-87.5) | 88.2 (87.6-88.8) | 91.5 (90.9-92.0) | 86.7 (86.3-87.1) |
| **Adjusted mean percentage virally suppressed*** |  |  |  |  |  |
| Retail dispensing | 81.9 (79.5-84.1) | 85.8 (84.0-87.5) | 92.1 (90.0-93.8) | 91.7 (90.4-92.9) | 89.4 (88.4-90.3) |
| Courier delivery | 86.2 (84.2-88.0) | 87.1 (85.3-88.7) | 91.9 (89.7-93.7) | 92.5 (91.3-93.6) | 90.5 (89.6-91.3) |

Participants could contribute multiple viral load tests. Generalized estimating equations were used to account for repeated viral load measurements from the same individual. Abbreviations: ART, antiretroviral therapy; OR, odds ratio; *Adjusted for history of mental illness, sex, age, ART regimen, and calendar year (overall analysis only).

Supplemental Table 4. Unadjusted and adjusted odds ratios for viral suppression (<400 RNA viral load copies per mL), comparing men on courier ART delivery to those on retail ART dispensing, and crude and adjusted mean probabilities of viral suppression for both ART distribution methods. Results are provided by calendar period and overall.

|  | **Calendar period** | | | | **Overall** |
| --- | --- | --- | --- | --- | --- |
|  | 2011-2013 | 2014-2016 | 2017-2019 | 2020-2022 |  |
| **Unadjusted OR for viral suppression** |  |  |  |  |  |
| Retail dispensing | 1 | 1 | 1 | 1 | 1 |
| Courier delivery | 1.26 (1.13-1.41) | 1.05 (0.97-1.14) | 0.92 (0.87-0.97) | 0.98 (0.92-1.05) | 0.98 (0.94-1.02) |
| **Adjusted OR for viral suppression*** |  |  |  |  |  |
| Retail dispensing | 1 | 1 | 1 | 1 | 1 |
| Courier delivery | 1.25 (1.12-1.40) | 1.00 (0.92-1.08) | 0.91 (0.86-0.97) | 0.97 (0.91-1.04) | 1.00 (0.96-1.04) |
| **Crude percentage virally suppressed** |  |  |  |  |  |
| Retail dispensing | 78.1 (76.7-79.4) | 86.6 (86.0-87.3) | 86.9 (86.4-87.4) | 88.7 (88.2-89.1) | 85.5 (85.1-85.9) |
| Courier delivery | 81.8 (80.5-83.0) | 87.2 (86.5-87.9) | 85.9 (85.3-86.4) | 88.5 (87.9-89.0) | 85.3 (84.8-85.7) |
| **Adjusted mean percentage virally suppressed*** |  |  |  |  |  |
| Retail dispensing | 80.4 (64.0-90.4) | 85.1 (83.9-86.2) | 90.1 (87.8-92.1) | 87.6 (86.8-88.3) | 88.1 (87.3-88.9) |
| Courier delivery | 83.6 (69.0-92.2) | 85.1 (83.8-86.2) | 89.3 (86.7-91.4) | 87.3 (86.4-88.1) | 88.1 (87.3-88.9) |

Participants could contribute multiple viral load tests. Generalized estimating equations were used to account for repeated viral load measurements from the same individual. Abbreviations: ART, antiretroviral therapy; OR, odds ratio; *Adjusted for history of mental illness, age, ART regimen, calendar year (overall analysis only), and medical scheme.

Supplemental Table 5. Unadjusted and adjusted odds ratios for viral suppression (<400 RNA viral load copies per mL), comparing women on courier ART delivery to those on retail ART dispensing, and crude and adjusted mean probabilities of viral suppression for both ART distribution methods. Results are provided by calendar period and overall.

|  | **Calendar period** | | | | **Overall** |
| --- | --- | --- | --- | --- | --- |
|  | 2011-2013 | 2014-2016 | 2017-2019 | 2020-2022 |  |
| **Unadjusted OR for viral suppression** |  |  |  |  |  |
| Retail dispensing | 1 | 1 | 1 | 1 | 1 |
| Courier delivery | 1.59 (1.44-1.75) | 1.23 (1.13-1.34) | 1.03 (0.97-1.09) | 1.09 (1.01-1.17) | 1.06 (1.01-1.10) |
| **Adjusted OR for viral suppression*** |  |  |  |  |  |
| Retail dispensing | 1 | 1 | 1 | 1 | 1 |
| Courier delivery | 1.44 (1.30-1.59) | 1.12 (1.03-1.22) | 1.00 (0.94-1.06) | 1.06 (0.99-1.14) | 1.10 (1.06-1.15) |
| **Crude percentage virally suppressed** |  |  |  |  |  |
| Retail dispensing | 80.2 (79.0-81.3) | 88.7 (88.1-89.2) | 90.1 (89.7-90.4) | 92.0 (91.6-92.3) | 89.1 (88.8-89.4) |
| Courier delivery | 86.5 (85.7-87.3) | 90.6 (90.0-91.2) | 90.4 (89.9-90.8) | 92.6 (92.2-92.9) | 89.6 (89.3-90.0) |
| **Adjusted mean percentage virally suppressed*** |  |  |  |  |  |
| Retail dispensing | 81.3 (75.5-86.1) | 88.8 (87.7-89.8) | 93.1 (91.5-94.5) | 92.4 (91.7-93.0) | 91.7 (91.0-92.3) |
| Courier delivery | 86.3 (81.7-89.9) | 89.9 (88.8-90.9) | 93.1 (91.4-94.5) | 92.8 (92.1-93.4) | 92.4 (91.8-93.0) |

Participants could contribute multiple viral load tests. Generalized estimating equations were used to account for repeated viral load measurements from the same individual. Abbreviations: ART, antiretroviral therapy; OR, odds ratio; *Adjusted for history of mental illness, age, ART regimen, calendar year (overall analysis only), and medical scheme.

Supplemental Table 6: Unadjusted and adjusted odds ratios for viral suppression (<400 RNA viral load copies per mL), comparing individuals on courier ART delivery to those on retail ART dispensing, and crude and adjusted mean probabilities of viral suppression for both ART distribution methods. Results are provided by calendar period and overall. The ART delivery type variable has been lagged by 6 months.

|  | **Calendar period** | | | | **Overall** |
| --- | --- | --- | --- | --- | --- |
|  | 2011-2013 | 2014-2016 | 2017-2019 | 2020-2022 |  |
| **Unadjusted OR for viral suppression** |  |  |  |  |  |
| Retail dispensing | 1 | 1 | 1 | 1 | 1 |
| Courier delivery | 1.48 (1.35-1.61) | 1.10 (1.03-1.18) | 0.92 (0.88-0.96) | 0.99 (0.94-1.04) | 0.98 (0.95-1.01) |
| **Adjusted OR for viral suppression*** |  |  |  |  |  |
| Retail dispensing | 1 | 1 | 1 | 1 | 1 |
| Courier delivery | 1.36 (1.24-1.48) | 1.00 (0.94-1.08) | 0.90 (0.86-0.93) | 0.97 (0.92-1.02) | 0.99 (0.96-1.02) |
| **Crude percentage virally suppressed** |  |  |  |  |  |
| Retail dispensing | 80.7 (79.8-81.7) | 88.1 (87.6-88.6) | 89.2 (88.9-89.5) | 90.8 (90.5-91.1) | 88.3 (88.0-88.5) |
| Courier delivery | 86.1 (85.3-86.8) | 89.1 (88.5-89.6) | 88.3 (88.0-88.7) | 90.7 (90.4-91.0) | 88.0 (87.8-88.3) |
| **Adjusted mean percentage virally suppressed*** |  |  |  |  |  |
| Retail dispensing | 81.4 (74.8-86.6) | 89.4 (87.9-90.7) | 92.2 (90.5-93.6) | 91.6 (90.8-92.3) | 90.4 (89.9-91.0) |
| Courier delivery | 85.6 (80.1-89.8) | 89.4 (88.0-90.8) | 91.4 (89.5-92.9) | 91.4 (90.5-92.1) | 90.4 (89.8-90.9) |

Participants could contribute multiple viral load tests. Generalized estimating equations were used to account for repeated viral load measurements from the same individual. Abbreviations: ART, antiretroviral therapy; OR, odds ratio; *Adjusted for history of mental illness, sex, age, ART regimen, calendar year (overall analysis only), and medical scheme.

Supplemental Table 7: Unadjusted and adjusted odds ratios for viral suppression (<400 RNA viral load copies per mL), comparing individuals on courier ART delivery to those on retail ART dispensing, and crude and adjusted mean probabilities of viral suppression for both ART distribution methods. Results are provided by calendar period and overall. The delivery type variable has been lagged by 12 months.

|  | **Calendar period** | | | | **Overall** |
| --- | --- | --- | --- | --- | --- |
|  | 2011-2013 | 2014-2016 | 2017-2019 | 2020-2022 |  |
| **Unadjusted OR for viral suppression** |  |  |  |  |  |
| Retail dispensing | 1 | 1 | 1 | 1 | 1 |
| Courier delivery | 1.45 (1.31-1.61) | 1.13 (1.04-1.23) | 0.93 (0.89-0.97) | 0.97 (0.93-1.02) | 0.97 (0.94-1.01) |
| **Adjusted OR for viral suppression*** |  |  |  |  |  |
| Retail dispensing | 1 | 1 | 1 | 1 | 1 |
| Courier delivery | 1.33 (1.20-1.49) | 1.06 (0.98-1.16) | 0.90 (0.86-0.94) | 0.96 (0.91-1.01) | 0.97 (0.94-1.01) |
| **Crude percentage virally suppressed** |  |  |  |  |  |
| Retail dispensing | 81.6 (80.5-82.6) | 85.2 (84.4-85.9) | 89.3 (89.0-89.6) | 90.9 (90.6-91.2) | 88.6 (88.3-88.8) |
| Courier delivery | 86.5 (85.5-87.5) | 86.7 (85.8-87.5) | 88.6 (88.2-88.9) | 90.7 (90.4-91.0) | 88.3 (88.0-88.6) |
| **Adjusted mean percentage virally suppressed*** |  |  |  |  |  |
| Retail dispensing | 82.6 (75.0-88.2) | 84.8 (81.7-87.4) | 90.4 (88.6-92.0) | 91.5 (90.7-92.2) | 89.8 (89.1-90.4) |
| Courier delivery | 86.3 (80.0-90.9) | 85.5 (82.6-88.0) | 89.5 (87.5-91.2) | 91.1 (90.3-91.9) | 89.5 (88.8-90.2) |

Participants could contribute multiple viral load tests. Generalized estimating equations were used to account for repeated viral load measurements from the same individual. Abbreviations: ART, antiretroviral therapy; OR, odds ratio; *Adjusted for history of mental illness, sex, age, ART regimen, calendar year (overall analysis only), and medical scheme.

Supplemental Table 8: Unadjusted and adjusted odds ratios for viral suppression (<50 RNA viral load copies per mL), comparing individuals on courier ART delivery to those on retail ART dispensing, and crude and adjusted mean probabilities of viral suppression for both ART distribution methods. Results are provided by calendar period and overall.

|  | **Calendar period** | | | | **Overall** |
| --- | --- | --- | --- | --- | --- |
|  | 2011-2013 | 2014-2016 | 2017-2019 | 2020-2022 |  |
| **Unadjusted OR for viral suppression** |  |  |  |  |  |
| Retail dispensing | 1 | 1 | 1 | 1 | 1 |
| Courier delivery | 1.36 (1.28-1.44) | 1.10 (1.05-1.16) | 0.97 (0.94-1.00) | 0.99 (0.95-1.02) | 1.00 (0.98-1.02) |
| **Adjusted OR for viral suppression*** |  |  |  |  |  |
| Retail dispensing | 1 | 1 | 1 | 1 | 1 |
| Courier delivery | 1.26 (1.19-1.34) | 1.04 (0.99-1.09) | 0.95 (0.92-0.98) | 0.98 (0.94-1.01) | 1.03 (1.01-1.05) |
| **Crude percentage virally suppressed** |  |  |  |  |  |
| Retail dispensing | 66.6 (65.6-67.6) | 80.4 (79.9-80.9) | 81.0 (80.7-81.4) | 83.3 (83.0-83.6) | 79.4 (79.1-79.7) |
| Courier delivery | 73.0 (72.2-73.8) | 81.9 (81.4-82.4) | 80.6 (80.2-81.0) | 83.1 (82.7-83.5) | 79.4 (79.1-79.7) |
| **Adjusted mean percentage virally suppressed*** |  |  |  |  |  |
| Retail dispensing | 63.4 (57.8-68.7) | 79.6 (78.1-81.0) | 81.0 (79.7-82.3) | 81.8 (80.9-82.7) | 78.8 (78.0-79.5) |
| Courier delivery | 68.6 (63.3-73.4) | 80.2 (78.7-81.6) | 80.3 (78.9-81.6) | 81.5 (80.5-82.4) | 79.3 (78.5-80.0) |

Participants could contribute multiple viral load tests. Generalized estimating equations were used to account for repeated viral load measurements from the same individual. Abbreviations: ART, antiretroviral therapy; OR, odds ratio; *Adjusted for history of mental illness, sex, age, ART regimen, calendar year (overall analysis only), and medical scheme.

Supplemental Table 9: Unadjusted and adjusted odds ratios for viral suppression (<1000 RNA viral load copies per mL), comparing individuals on courier ART delivery to those on retail ART dispensing, and crude and adjusted mean probabilities of viral suppression for both ART distribution methods. Results are provided by calendar period and overall.

|  | **Calendar period** | | | | **Overall** |
| --- | --- | --- | --- | --- | --- |
|  | 2011-2013 | 2014-2016 | 2017-2019 | 2020-2022 |  |
| **Unadjusted OR for viral suppression** |  |  |  |  |  |
| Retail dispensing | 1 | 1 | 1 | 1 | 1 |
| Courier delivery | 1.49 (1.38-1.61) | 1.14 (1.07-1.22) | 0.97 (0.93-1.01) | 1.04 (0.99-1.09) | 1.02 (0.99-1.06) |
| **Adjusted OR for viral suppression*** |  |  |  |  |  |
| Retail dispensing | 1 | 1 | 1 | 1 | 1 |
| Courier delivery | 1.39 (1.28-1.50) | 1.06 (1.00-1.13) | 0.96 (0.91-1.00) | 1.03 (0.97-1.08) | 1.05 (1.02-1.08) |
| **Crude percentage virally suppressed** |  |  |  |  |  |
| Retail dispensing | 81.7 (80.9-82.6) | 89.5 (89.1-89.9) | 90.3 (90.0-90.5) | 92.0 (91.7-92.2) | 89.3 (89.1-89.5) |
| Courier delivery | 87.0 (86.3-87.6) | 90.7 (90.2-91.1) | 90.0 (89.7-90.3) | 92.3 (92.0-92.6) | 89.5 (89.2-89.7) |
| **Adjusted mean percentage virally suppressed*** |  |  |  |  |  |
| Retail dispensing | 84.1 (78.9-88.2) | 90.6 (89.4-91.6) | 94.2 (92.9-95.3) | 93.1 (92.4-93.7) | 92.3 (91.8-92.8) |
| Courier delivery | 88.0 (83.8-91.2) | 91.1 (89.9-92.1) | 93.9 (92.5-95.1) | 93.2 (92.5-93.9) | 92.7 (92.2-93.1) |

Participants could contribute multiple viral load tests. Generalized estimating equations were used to account for repeated viral load measurements from the same individual. Abbreviations: ART, antiretroviral therapy; OR, odds ratio; *Adjusted for history of mental illness, sex, age, ART regimen, calendar year (overall analysis only), and medical scheme.
